# Palliative care perceptions and well-being in nurses across healthcare settings: a comparative study from Reggio Emilia, Northern Italy

**DOI:** 10.1186/s12904-025-01911-2

**Published:** 2025-10-21

**Authors:** Chiara Bevini, Riccardo Mazzoli, Vanessa E. Privitera, Mirta Rocchi, Lucia Palandri, Anna Laura Santunione, Tommaso Filippini

**Affiliations:** 1 Environmental, Genetic and Nutritional Epidemiology Research Center (CREAGEN), Department of Biomedical, Metabolic and Neural Sciences, University of Modena and Reggio Emilia, Modena, Italy; 2 Department of Biomedical, Metabolic and Neural Sciences, University of Modena and Reggio Emilia, Modena, Italy; 3 Hospice ′Casa Madonna dell′Uliveto′, Reggio Emilia, Italy; 4 Legal Medicine, Department of Biomedical, Metabolic and Neural Sciences, University of Modena and Reggio Emilia, Modena, Italy; 5 School of Public Health, University of California Berkeley, Berkeley, CA USA

**Keywords:** Emotional distress, Home care, Hospice care, Hospital care, Nurses, Palliative care

## Abstract

**Background:**

Nurses play a crucial role in palliative care. They are employed across all settings including home care and hospitals, with potential differences in the perception of care, emotional burden and coping strategies. This study aims to explore the experience of nurses within the palliative care network of an Italian province.

**Methods:**

From January to March 2024, we carried out a tailored survey on nurses from three palliative care settings in Reggio Emilia (Northern Italy), namely home care, hospice and hospital ward. We investigated emotional and occupational experience, knowledge of palliative care and organizational challenges.

**Results:**

The study included 39 nurses, nearly 90% of whom female. Of these, 20 worked in hospices, 10 in hospital wards and 9 in home care. In relation to occupational issues, most participants believed that newly-graduated nurses need additional training before working independently in both hospital ward and home care. Home care seemed adequate to palliative care demands for most participants, who nonetheless remained skeptical about work in hospice. Major emotional challenges included supporting patients’ families and feeling alone during emergencies or during sensitive communication, especially in home care. Emotional support was reported as adequate by 61.5% of nurses. However, 35.9% felt it was insufficient, while two thirds of participants admitted to having cried in front of patients. Almost all nurses frequently witnessed patients’ deaths and noted that patients’ preferences for their place of death were sometimes overruled by caregivers or doctors.

**Conclusion:**

Palliative care nursing requires strong emotional resilience, effective communication and comprehensive clinical training. This is true regardless of the setting. Our findings suggest that nurses in home care may need additional organizational support to manage emergencies and reduce emotional stress. This could improve quality of care for patients as well as caregivers, and reduce burnout risk in healthcare professionals in the palliative care network.

**Supplementary Information:**

The online version contains supplementary material available at 10.1186/s12904-025-01911-2.

## Background

Palliative care originally dates back to the work of physician, nurse and several other health care professionals since the 1960 s [1, 2], and it has evolved significantly in concept, scope and timing of intervention [3]. Uncertainty in the definition of palliative care, however, has generated insecurity among healthcare workers and policymakers [4]. Initially defined by the World Health Organization (WHO) in 1990 as care for patients with incurable diseases [5], it has since expanded firstly to life-threatening conditions from a public health perspective [6] and later to all health-related suffering across ages. This shift is reflected by the International Association for Hospice and Palliative Care (IAHPC), which stresses the need for holistic care to improve quality of life for patients suffering from severe illness, especially of those near the end of life, their families and caregivers [7].

Italy introduced regulatory provisions to guarantee access to palliative care and pain therapy in all care settings with Law 38/2010, providing an individual care plan for the patient and the family. This aimed at guaranteeing dignity and autonomy, protection and promotion of quality of life and adequate health and social welfare support. Different models were defined and Networks of Palliative Care services have been implemented to ensure continuity of care [8].

Local Palliative Care Networks are an essential feature of effective palliative care in that these allow for collaboration among hospitals, homes and hospices [9]. Nurses play a pivotal role in these networks, where they liaise with other healthcare professionals, patients and families [10]. Their specialized competence, outlined in documents such as the Italian Palliative Nursing Core Competence [8] and European Palliative Care framework [11], includes ethical, clinical, communication, psychosocial and teamwork skills.

Many studies have investigated the role of nurses in palliative care. Almost all of them focus primarily on home care, followed by other settings such as hospitals [12]. Nurses working in palliative care are almost constantly exposed to difficult situations, death and feelings of emotional conflict, with studies indicating a high incidence of burnout, estimated between 24% and 30% [13]. Despite these challenges, the collaborative nature of palliative care, involving general practitioners (GPs), specialists and other nurses, fosters steady relationships that make it both exhausting and fulfilling [14].

Given the complexity of palliative care and the different care settings, this study aims to explore nurses’ diverse experiences across hospice, home care and hospital ward in Reggio Emilia (Northern Italy), in order to describe the specific challenges faced by palliative care nursing in different settings, to identify their coping strategies, and to address and prevent discomfort associated with the work environment.

## Methods

### Study objective, population and settings

We conducted our investigation between January and March 2024 in three settings from the Palliative Care Network of the Reggio Emilia province.

In order to assess emotional challenges, coping mechanisms, and perspectives from palliative care nurses, we implemented a tailored survey to identify potential differences among professionals working in hospices, home care and hospital ward. More specifically, we interviewed nurses from three settings: (i) two home nursing services, located in Scandiano and Reggio Emilia, both specialized in palliative care oncology; (ii) two hospital wards (Oncological Medicine and Hematology), both at the Oncological and Hematological Center (CORE) in Reggio Emilia; (iii) two hospices, namely ‘Casa Madonna dell’Uliveto’ and ‘Guastalla Hospice’, in Albinea and Guastalla.

All the participants were nurses working exclusively in one of the palliative care facilities/network in outpatient and inpatient care, with no concurrent or rotational duties across the different settings during the study period. No nursing students, auxiliary staff, or non-licensed personnel were included.

### Data collection

The study team including nurses, physicians and researchers in palliative care, health education and public health designed a questionnaire comprising 20 multiple-choice questions. Of these, three were subdivided into additional sub-questions. Moreover, the questionnaire included two open-ended questions. Each question addressed a specific aspect through a five-point Likert scale: emotional experience of healthcare providers; occupational experience and day-to-day activities; knowledge of medical and palliative care, and their importance in different care settings; and challenges in organization and training. Furthermore, we collected some demographical variables including gender, age, work role and years of service. Full questionnaire is reported in Supplementary methods. 

The questionnaire was implemented through the Google Forms platform (Google LLC), and was lawfully distributed via email to nursing coordinators from the selected study settings, who then forwarded it to staff members. Participants completed the form anonymously to ensure impartiality and seek honest feedback. In addition, in order to guarantee participants’ privacy and prevent identification of study subjects in light of the low number of professionals in some settings, we collected aggregated data about participants’ demographic variables. In more detail, we categorized age into five-year groups in order to avoid personal identification of participants. All participants provided the consent to participate before the completion of the questionnaire. Approval from the Ethics Committee was waived due to the use of entirely anonymous and aggregated data according to Italian law and guidelines from the Emilia Romagna Region (PG/2020/220858 of March 13, 2020) [15]. Out of 63 nurses working in hospice (*N* = 28), home care (*N* = 11), and hospital ward (*N* = 24) respectively, we gathered 39 questionnaires with an overall response rate of 61.9%, higher in home care (90.9%), followed by hospice (71.4%) and hospital ward (37.5%).

### Data analysis

We carried out a descriptive evaluation of data collected using absolute and relative frequencies, calculated as percentages across the entire sample and stratified by workplace setting, to describe and compare healthcare professionals’ responses. Data analysis was conducted using the statistical package Stata-18.0 (Stata Corp., College Station, TX, USA, 2023). We conducted a content analysis of open-ended responses identifying recurrent words and expressions, and categorizing them into conceptual domains relevant to palliative care practice.

## Results

Table 1 shows the socio-demographic characteristics of the study participants. The majority of respondents worked in hospices (*n* = 20). Female nurses amounted to nearly 90% of the sample, with male respondents working exclusively in hospice care. The predominant age groups were 25–35 and 45–55 years, while the years of service were equally distributed across settings.

**Table 1:** Socio-demographic and work-related characteristics of study participants

	Hospice	Home care	Hospital ward	Total
	*N* (%)	*N* (%)	*N* (%)	*N* (%)
All participants	20 (51.3)	10 (25.6)	9 (23.1)	30 (100)
Gender				
Females	16 (41.0)	10 (25.6)	9 (23.1)	35 (89.7)
Males	4 (10.3)	0 (0.0)	0 (0.0)	4 (10.3)
Age (years)				
25–35	5 (12.8)	3 (7.7)	5 (12.8)	13 (33.3)
35–45	5 (12.8)	1 (2.6)	3 (7.7)	9 (23.1)
45–55	9 (23.1)	5 (12.8)	1 (2.6)	15 (38.5)
> 55	1 (2.6)	1 (2.6)	0 (0.0)	2 (5.1)
Years of service				
< 5	5 (12.8)	3 (7.7)	2 (5.1)	10 (25.6)
5–10	4 (10.3)	0 (0.0)	6 (15.4)	10 (25.6)
10–20	5 (12.8)	4 (10.3)	1 (2.3)	10 (25.6)
> 20	6 (15.4)	3 (7.7)	0 (0.0)	9 (23.1)

With regards to the nurses’ opinion about palliative care work (Table 2), an overwhelming majority (87.2%) believed that newly-graduated hospital nurses should not work independently in palliative care. This opinion was consistent across all settings, with hospice nurses being the most skeptical. Similarly, there was a clear consensus (94.9%) that additional experience or training is required before newly-graduated home care palliative nurses can work independently. The vast majority (94.9%) of nurses across all settings believed that their departments or sectors had sufficient resources to deliver effective levels of care. Moreover, most nurses (84.6%) stated that home care can met palliative care demands, although a minority of hospice nurses (12.8%) disagreed.

**Table 2:** Nurses’ opinions on the ability to work independently, resource adequacy, and capacity to meet demands in palliative care

	Hospice	Home care	Hospital ward	Total
	*N* (%)	*N* (%)	*N* (%)	*N* (%)
Independent work in hospital for newly-graduated nurses				
No	19 (48.7)	8 (20.5)	7 (17.9)	34 (87.2)
Yes	1 (2.6)	2 (5.1)	2 (5.13)	5 (12.8)
Independent work in home care for newly-graduated nurses				
No	20 (51.3)	9 (23.1)	8 (20.5)	37 (94.9)
Yes	0 (0.0)	1 (2.6)	1 (2.6)	2 (5.1)
Availability of sufficient resources				
No	0 (0.0)	1 (2.6)	1 (2.6)	2 (5.1)
Yes	20 (51.3)	9 (23.1)	8 (20.5)	37 (94.9)
Suitability of home care for palliative care				
No	5 (12.8)	0 (0.0)	1 (2.6)	6 (15.4)
Yes	15 (38.5)	10 (20.6)	8 (20.5)	33 (84.6)

Nurses’ answers with regard to professional opinions and self-assessment of training are presented in Table 3. In detail, half of them believed that home nursing care and joint access to palliative care physicians and GPs were not sufficient to assist patients at the end of life. Hospice nurses were the most skeptical, while hospital nurses were more optimistic. On the other hand, there was a strong consensus (84.6%) that palliative care should be proposed in the initial phase, alongside active treatment. Nearly all nurses (92.3%) thought that supporting patients’ families is very challenging, while the remaining nurses found it moderately challenging. Nonetheless, most nurses (86.8%) reported feeling adequately prepared to communicate with patients and their families about approaching death, although a small percentage (13.2%) felt they were not. Nurses had mixed opinions on adequate training in clinical practice, with nearly half of them feeling ‘sufficiently trained’, and the other half ‘somewhat trained’. The majority of participants (51.3%) felt moderately prepared to communication, while a smaller group (33.3%) felt fully prepared. When considering palliative care as a career path, the majority (79.5%) of respondents believed it to be a suitable career choice, while a minority (20.5%) feel a degree of uncertainty. It is worth noting that none of the participants thought that the career did not suit them.

**Table 3:** Nurses’ professional opinions and self-assessed training/preparation

	Hospice	Home care	Hospital ward	Total
	*N* (%)	*N* (%)	*N* (%)	*N* (%)
Adequacy of home nursing care and joint access to palliative care physicians and general practitioners				
No	12 (30.8)	5 (12.8)	3 (7.7)	20 (51.3)
Yes	8 (20.5)	5 (12.8)	6 (15.4)	19 (48.7)
Appropriate stage to propose palliative care				
Early stage with active treatment	18 (46.2)	8 (20.5)	7 (17.9)	33 (84.6)
When active treatments are no longer effective	2 (5.1)	2 (5.1)	2 (5.1)	6 (15.4)
Terminal phase of the disease	0 (0.0)	0 (0.0)	0 (0.0)	0 (0.0)
Difficulty in assisting the patient’s family				
Not at all	0 (0.0)	0 (0.0)	0 (0.0)	0 (0.0)
A little	0 (0.0)	0 (0.0)	0 (0.0)	0 (0.0)
Somewhat	1 (2.6)	0 (0.0)	2 (5.1)	3 (7.7)
Much	19 (48.7)	9 (23.1)	7 (17.9)	35 (89.7)
A great deal	0 (0.0)	1 (2.6)	0 (0.0)	1 (2.6)
Preparedness to communicate approaching death to patient and family				
No	1 (2.6)	1 (2.6)	3 (7.9)	5 (13.2)
Yes	19 (50.0)	8 (21.1)	6 (15.8)	33 (86.8)
Adequate training in clinical practice				
Strongly disagree	0 (0.0)	0 (0.0)	0 (0.0)	0 (0.0)
Disagree	0 (0.0)	1 (2.6)	0 (0.0)	1 (2.6)
Agree	9 (23.1)	6 (15.4)	4 (10.3)	19 (48.7)
Strongly agree	11 (28.2)	3 (7.7)	5 (12.8)	19 (48.7)
Adequate training in communication				
Strongly disagree	0 (0.0)	0 (0.0)	0 (0.0)	0 (0.0)
Disagree	3 (7.7)	2 (5.1)	1 (2.6)	6 (15.4)
Agree	8 (20.5)	7 (17.9)	5 (12.8)	20 (51.3)
Strongly agree	9 (23.1)	1 (2.6)	3 (7.7)	13 (33.3)
Palliative care as the right career fit				
Disagree	0 (0.0)	0 (0.0)	0 (0.0)	0 (0.0)
Unsure	2 (5.1)	3 (7.6)	3 (7.7)	8 (20.5)
Agree	18 (46.2)	7 (17.9)	6 (15.4)	31 (79.5)

Table 4 shows the responses related to emotional demands of nurses working in palliative care services. Most nurses (56.4%) felt a significant amount of responsibility when starting palliative sedation, with hospice nurses reporting this feeling the most, while another substantial portion (30.8%) acknowledged a moderate level of responsibility. The majority of nurses (87.2%) reported that they managed to remain calm during their first palliative emergency. This is the case despite the fact that two-thirds (66.7%) of nurses stated they have felt alone in stressful situations, with home care nurses most likely to experience isolation. A large majority (87.2%) have experienced excessive involvement, with a high percentage of nurses (66.7%) confessing to having cried in front of patients. With regards to psychological support, a majority (61.5%) reported receiving adequate psychological help from their institution, while a sizeable minority (35.9%) did not. Most nurses (41.0%) reported feeling mentally tired at the end of their shifts. In particular, 35.9% felt both physically and mentally tired, while home care nurses were more likely to report mental fatigue.

**Table 4:** Nurses’ emotional involvement and emotional burden

	Hospice	Home care	Hospital ward	Total
	*N* (%)	*N* (%)	*N* (%)	*N* (%)
Level of responsibility when administering palliative sedation				
Not at all	0 (0.0)	0 (0.0)	0 (0.0)	0 (0.0)
A little	3 (7.7)	2 (5.1)	0 (0.0)	5 (12.8)
Some	6 (15.4)	4 (10.3)	2 (5.1)	12 (30.8)
Much	11 (28.2)	4 (10.3)	7 (17.9)	22 (56.4)
A great deal	0 (0.0)	0 (0.0)	0 (0.0)	0 (0.0)
Composure during the first palliative emergency				
No	2 (5.1)	0 (0.0)	1 (2.6)	3 (7.7)
Not yet experienced	0 (0.0)	1 (2.6)	1 (2.6)	2 (5.1)
Yes	18 (46.2)	9 (23.1)	7 (17.9)	34 (87.2)
Experience of excessive involvement (e.g., feeling that what happened to the patient could happen to you or your loved ones)				
No	3 (7.7)	1 (2.6)	1 (2.6)	5 (12.8)
Yes	17 (43.6)	9 (23.1)	8 (20.5)	34 (87.2)
Loneliness in dealing with a delicate situation				
No	9 (23.1)	0 (0.0)	4 (10.3)	13 (33.3)
Yes	11 (28.2)	10 (25.6)	5 (12.8)	26 (66.7)
Instances of crying (for any reason) in front of a patient				
No	4 (10.3)	5 (12.8)	4 (10.3)	13 (33.3)
Yes	16 (41.0)	5 (12.8)	5 (12.8)	26 (66.7)
Adequate and specific psychological support provided by institutions				
No	4 (10.3)	6 (15.4)	4 (10.3)	14 (35.9)
Yes	15 (38.5)	4 (10.3)	5 (12.8)	24 (61.5)
Not reported	1 (2.6)	0 (0.0)	0 (0.0)	1 (2.6)
State of tiredness at the end of workdays				
Physically and mentally	10 (25.6)	1 (2.6)	3 (7.7)	14 (35.9)
Physically only	3 (7.7)	1 (2.6)	2 (5.1)	6 (15.4)
Mentally only	5 (12.8)	7 (17.9)	4 (10.3)	16 (41.0)
Not particularly	2 (5.1)	1 (2.6)	0 (0.0)	3 (7.7)

Table 5 provides insights into nurses’ experiences of patients’ end-of-life. The vast majority of nurses (94.9%) across all settings witnessed patients’ deaths, with only one nurse in home care and one in a hospital ward reporting otherwise. Most nurses (41.0%) frequently handle imminent end-of-life scenarios alongside colleagues (‘always’ and ‘often’). This was especially evident among hospice nurses (28.2%), while it is less common among home care nurses. Every nurse reports having acknowledged the patient’s preference for their place of death. Nearly all (97.4%) encountered cases where caregivers opposed the patient’s preference, while a large portion (64.1%) observed instances of doctors overruling the patient’s decision. Many nurses (69.2%), especially those in hospice settings, reported instances where the patient’s dignity was not respected during end-of-life care. A substantial proportion of nurses (64.1%) noted occasions where optimal pain control was not consistently guaranteed. This trend can be seen for both hospice and home care nurses, while it is notable that five in nine hospital nurses stated that pain management was consistently provided.

**Table 5:** Nurses’ experience of patients’ death, challenges and ethical considerations

	Hospice	Home care	Hospital ward	Total
	*N* (%)	*N* (%)	*N* (%)	*N* (%)
Witnessing patients dying				
No	0 (0.0)	1 (2.6)	1 (2.6)	2 (5.1)
Yes	20 (51.3)	9 (23.1)	8 (20.5)	37 (94.9)
Managing a patient’s imminent end of life with colleagues (nurses, assistants, physicians) present				
Always	11 (28.2)	1 (2.6)	4 (10.3)	16 (41.0)
Often	6 (15.4)	0 (0.0)	3 (7.7)	9 (23.1)
Sometimes	3 (7.7)	7 (17.9)	2 (5.1)	12 (30.8)
Rarely	0 (0.0)	2 (5.1)	0 (0.0)	2 (5.1)
Respect of the patient’s preference for their place of death (e.g., home, hospice)				
No	0 (0.0)	0 (0.0)	0 (0.0)	0 (0.0)
Yes	20 (51.3)	10 (25.6)	9 (23.1)	39 (100)
Caregivers opposition to the patient’s choice of place of death				
No	0 (0.0)	1 (2.6)	0 (0.0)	1 (2.6)
Yes	20 (51.3)	9 (23.1)	9 (23.1)	38 (97.4)
Doctors overruling the patient’s choice of place of death				
No	8 (20.5)	2 (5.1)	4 (10.3)	14 (35.9)
Yes	12 (30.8)	8 (20.5)	5 (12.8)	25 (64.1)
Instances of compromised patient dignity during end-of-life care				
No	4 (10.3)	4 (10.3)	4 (10.3)	12 (30.8)
Yes	16 (41.0)	6 (15.4)	5 (12.8)	27 (69.2)
Consistency in achieving maximum and adequate pain control across all care settings				
No	14 (35.9)	7 (17.9)	4 (10.3)	25 (64.1)
Yes	6 (15.4)	3 (7.7)	5 (12.8)	14 (35.9)

**Fig. 1:**
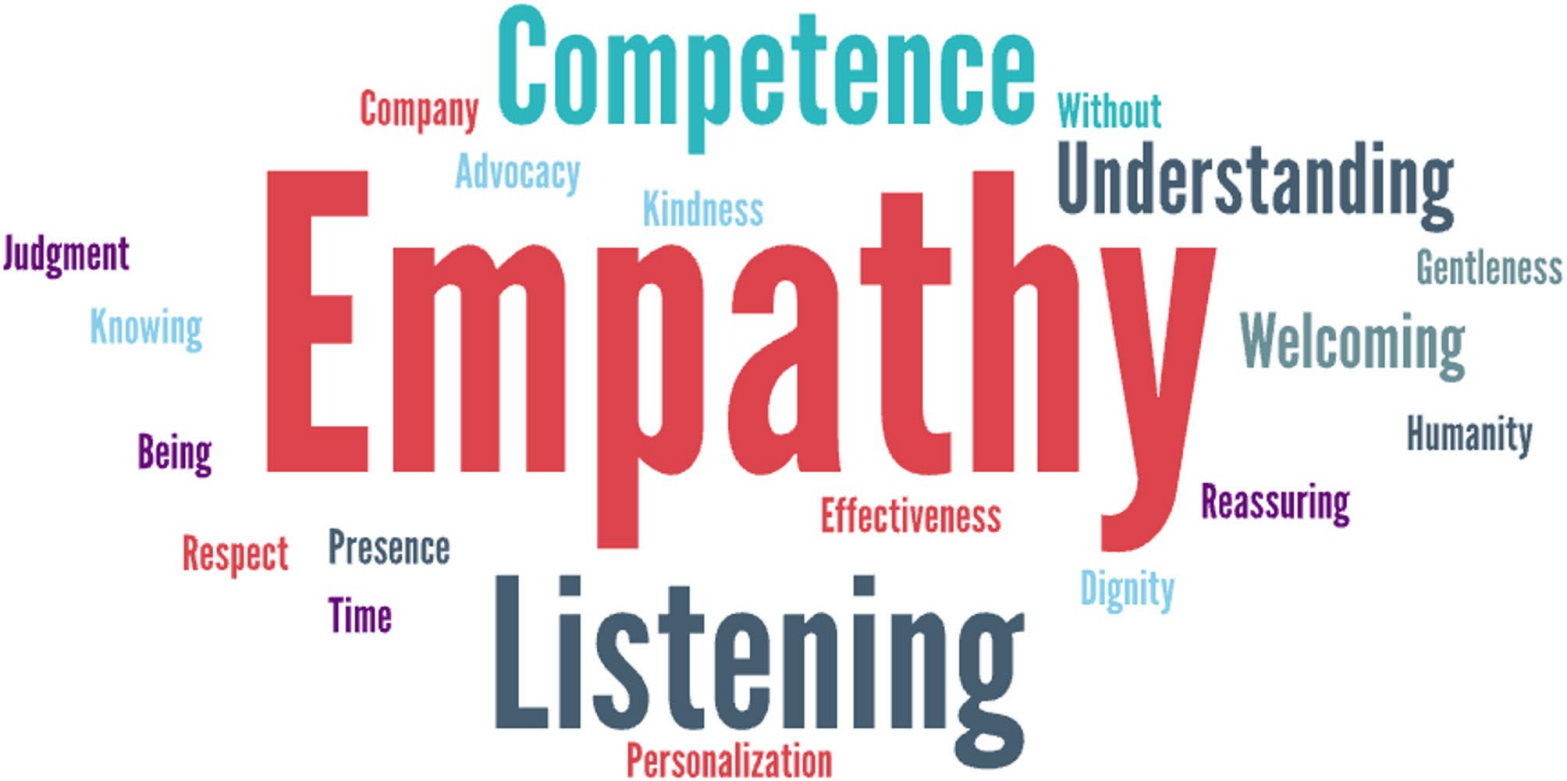
Word cloud created from asking nurses to describe palliative care in one word

When asked about essential attributes of palliative care nurses, common themes emerge: empathy and compassion, presence, communication and listening skills, and competence (Fig. 1 and Supplementary Table S1).

## Discussion

The findings from our study provide an overview of hospice, home care and hospital ward palliative care nurses’ perceptions and opinions. There was consensus that newly-graduated nurses in both hospital and home care settings needed additional experience or training before working independently in palliative care. Consistently with our findings, a review [16] highlighted the importance of professional support for these nurses, especially when undergoing strong emotional experiences such as patient’s death. Other studies [17] found that newly-graduated nurses gained confidence when supervised and supported by experienced nurses.

Despite challenges, most participants believed their departments had enough resources for effective palliative care, and that home care could meet palliative care needs. This perception was consistent across various settings, despite most respondents not being home care workers. This result is in keeping with a study conducted in The Netherlands, which also reported high ratings for home palliative care [18]. However, other studies indicated insufficient personnel [19] and time constraints [20], suggesting variability in resource allocation and perception in different settings.

In terms of joint access to palliative care physicians and GPs, half of the nurses in our sample questioned its adequacy, with hospice nurses as the most skeptical and hospital nurses having a more optimistic outlook. The divided opinions on home nursing care and the joint access model reflect ongoing debates about optimal palliative care delivery, which varies widely in structure and effectiveness [21, 22]. Literature supports collaborative approaches involving palliative care specialists, GPs and nursing services for improved patient outcomes and satisfaction [23–26].

Despite differing opinions on joint access, most participants (84.6%) agreed on early integration of palliative care combined with active treatment. This is strongly supported by evidence, as it is shown to: improve patient quality of life and survival [27, 28], facilitate symptom control, increase patient’s and family’s satisfaction, and align with patient preferences [29].

While most nurses feel clinically trained to discuss death, a vast majority (92.3%) found supporting the patient’s family to be exceedingly challenging. This emphasizes the emotional demands of end-of-life care. Recent literature highlights the importance of effective communication and structured training programs, as these can improve healthcare professionals’ ability to conduct end-of-life conversations [30, 31]. Regular meetings, group debriefings, role-playing, simulations and experience sharing can help overcome perceived limitations. Integrating these practices into Italian palliative care nurse training can improve communication with patients and families and reduce healthcare providers’ mental strain.

A large portion of respondents had reservations about their career choice in palliative care, which may be an indicator of burnout. It is well documented that high burnout levels are associated with drop out of professionals from the field [32–35]. Most studies on this issue focus on institutional and non-home-based settings, while research in home care settings specifically found that supportive environments lead to lower burnout and better nurse retention [32]. Studies showed that home care nurses have frequent contact with colleagues, patients and caregivers, while hospital workers reported less colleague and psychosocial support. Nurses in both settings, however, face similar burnout risks [36, 37]. This chimes with our data, where a third of hospital ward nurses felt less committed to continuing their work in palliative care, however generally satisfied with their career choice.

The responsibility reported by all nurses during palliative sedation reflects the ethical and emotional weight of these decisions [38]. Emotional involvement is well-documented [39–42] and can lead to emotional exhaustion [40] and “compassion fatigue” [41, 42], a term specifically referring to the weariness experienced by caregivers constantly exposed to situations of suffering and terminality. While most participants received adequate emotional support, our study suggested that this support may not be universally provided or sufficient.

Our study found that hospice nurses reported higher physical and mental fatigue, possibly due to heavy workloads from personal hygiene assistance, patient mobilization, night shifts and frequent meetings. On the other hand, home care nurses primarily reported mental fatigue, mainly due to isolation, terminal symptoms and emergency management, and communication with patients and caregivers often unprepared for an unfavorable diagnosis [43]. Furthermore, unlike in hospice and hospital, there was no arrangement for a health and social care assistant to work with the home nursing care network. Our findings are consistent with Petean et al. (2016), who showed that home care nurses face more ethical dilemmas and carry more emotional burdens associated with patient deaths than hospital nurses, and they needed more support including bioethicists and psycho-oncologists within the multidisciplinary team [43]. Most nurses in our sample frequently manage end-of-life scenarios with colleagues, particularly in hospice settings, which is considered to be essential [44, 45]. When included in the interdisciplinary team, psycho-oncologists provide psychotherapeutic interventions to patients and healthcare workers to manage emotional distress, existential issues, and adjustment challenges [46, 47]. In particular, psycho-oncologists may help other interdisciplinary team members who are distressed about discussing death [48]. They may provide a guidance to assess patient readiness and preferences for conversations about end-of-life, and to allow for an open conversation about death within these boundaries [46]. Bioethicists are helpful navigate complex ethical dilemmas, such as end-of-life decision-making, patient rights, and informed consent, to ensure respectful, person-centered care that aligns with patient values. Several studies emphasized the relevance of specific approaches, like spiritual care interventions [49–51].

All nurses gave due consideration to patients’ preference for their place of death. Nearly all encountered caregiver opposition to patients’ choices and many observed doctors overruling patient decisions, especially in hospice settings. In light of the paramount and renowned importance of patient choice [52–54], this poses ethical questions about respecting patient autonomy and participation in decision-making. Reported lapses in pain control indicate gaps in achieving consistent, high-quality palliative care [55].

When asked for a key quality of palliative care nurses, the majority of our sample mentioned “empathy”, followed by an “ability to listen” and “competence”. Treglia et al. (2020) [56] argued that empathy is a skill that needs to be sharpened in that it leads to better outcomes and patient satisfaction. Training and emotional support for healthcare professionals should therefore be encouraged. Additionally, empathy between practitioners and patients reduces pain perception and increases pain control, as demonstrated in a study [57].

### Strengths and limitations

The strengths in our research include comparing nurses’ perceptions from different settings, namely hospices, home care and hospital ward, with responses from almost all Palliative Care Network settings from the province of Reggio Emilia. However, this may limit the scope for broader generalization from our findings. To begin with, the relatively small sample size limited the identification of some differences across the investigated domains. In addition, a predominance of female participants restricted gender-based analysis. Moreover, self-report data collection suggests that caution should be used in interpreting results. Future research could extend similar questionnaires to palliative care physicians or evaluate interventions for preventing burnout and emotional stress in palliative care workers.

## Conclusion

The collected data highlights the challenges faced by palliative care nursing in different settings. Management of emotions, effective communication strategies and optimal clinical training are the areas that require greater support, especially in home care setting. We also highlighted the need for additional staff including bioethicists and psycho-oncologists to assist multidisciplinary teams in managing emergencies and enhancing quality of care. Such support is essential to reducing the risk of emotional stress and facilitating discussion of the ethical dilemmas faced by healthcare providers when assisting individuals in the final stages of life.

## Supplementary Information

Supplementary material 1.

## Data Availability

All data generated or analyzed for this study are included in this published article and its supplementary information files.

## References

[1] Lutz S. The history of hospice and palliative care. Curr Probl Cancer. 2011;35(6):304–9. 22136703 10.1016/j.currproblcancer.2011.10.004

[2] Clark D. From margins to centre: a review of the history of palliative care in cancer. Lancet Oncol. 2007;8(5):430–8. 17466900 10.1016/S1470-2045(07)70138-9

[3] Wajid M, Rajkumar E, Romate J, George AJ, Lakshmi R, Simha S. Why is hospice care important? An exploration of its benefits for patients with terminal cancer. BMC Palliat Care. 2021;20(1):70. 34001076 10.1186/s12904-021-00757-8 PMC8130431

[4] Ryan S, Wong J, Chow R, Zimmermann C. Evolving definitions of palliative care: upstream migration or confusion? Curr Treat Options Oncol. 2020;21(3):20. 32048055 10.1007/s11864-020-0716-4

[5] WHO. Cancer pain relief and palliative care. Report of a WHO expert committee. World Health Organ Tech Rep Ser. 1990;804:1–75. 1702248

[6] Organization WH. National cancer control programmes: policies and managerial guidelines. World Health Organization; 2002.

[7] Radbruch L, De Lima L, Knaul F, Wenk R, Ali Z, Bhatnaghar S, Blanchard C, Bruera E, Buitrago R, Burla C, et al. Redefining palliative Care-A new Consensus-Based definition. J Pain Symptom Manage. 2020;60(4):754–64. 32387576 10.1016/j.jpainsymman.2020.04.027 PMC8096724

[8] Prandi C, Mastroianni C, D’Angelo D, Marson R, Malinverni E, Guarda M. Il core competence italiano dell’infermiere in cure palliative (Italian Palliative Nursing Core Competence). 2018. p. 116.

[9] Moroni L, Peruselli C, Fortini G, Orsi L, Bastianello S, Di Silvestre G, Rizzi B, Bonesi MG. Il modello organizzativo in cure palliative. Rivista Italiana Di Cure Palliat. 2019;21(4):248–52.

[10] Sekse RJT, Hunskår I, Ellingsen S. The nurse’s role in palliative care: A qualitative meta-synthesis. J Clin Nurs. 2018;27(1–2):e21–38. 28695651 10.1111/jocn.13912

[11] Core Palliative Care Research Competencies Framework. for Palliative Care Clinicians [https://eapcnet.eu/eapc-publications/] 10.1089/jpm.2023.0399 38010819

[12] Autelitano C, Bertocchi E, Artioli G, Alquati S, Tanzi S. The specialist palliative care nurses’ in an Italian hospital: role, competences, and activities. Acta Biomed. 2021;92(S2):e2021006. 33855987 10.23750/abm.v92iS2.11360 PMC8138805

[13] Gomez-Urquiza JL, Albendin-Garcia L, Velando-Soriano A, Ortega-Campos E, Ramirez-Baena L, Membrive-Jimenez MJ, Suleiman-Martos N. Burnout in palliative care Nurses, prevalence and risk factors: A systematic review with Meta-Analysis. Int J Environ Res Public Health. 2020;17(20):7672. 10.3390/ijerph17207672 PMC7589426 33096682

[14] Parola V, Coelho A, Sandgren A, Fernandes O, Apostolo J. Caring in palliative care: A phenomenological study of nurses’ lived experiences. J Hosp Palliat Nurs. 2018;20(2):180–6. 30063572 10.1097/NJH.0000000000000428

[15] Vinceti SR, Filippini T. Revising the legislation of ethics committees to ease biomedical research in humans across the world: lessons from the COVID-19 emergency. Acta Biomed. 2022;93(2):e2021579. 35546005 10.23750/abm.v93i2.12582 PMC9171886

[16] Zheng R, Lee SF, Bloomer MJ. How new graduate nurses experience patient death: A systematic review and qualitative meta-synthesis. Int J Nurs Stud. 2016;53:320–30. 26493131 10.1016/j.ijnurstu.2015.09.013

[17] Rolt L, Gillett K. Employing newly qualified nurses to work in hospices: A qualitative interview study. J Adv Nurs. 2020;76(7):1717–27. 32189368 10.1111/jan.14359

[18] Joren CY, de Veer AJE, de Groot K, Francke AL. Home care nurses more positive about the palliative care that is provided and their own competence than hospital nurses: a nationwide survey. BMC Palliat Care. 2021;20(1):170. 34711219 10.1186/s12904-021-00866-4 PMC8552607

[19] Diehl E, Rieger S, Letzel S, Schablon A, Nienhaus A, Escobar Pinzon LC, Dietz P. Burdens, resources, health and wellbeing of nurses working in general and specialised palliative care in Germany - results of a nationwide cross-sectional survey study. BMC Nurs. 2021;20(1):162. 34488742 10.1186/s12912-021-00687-z PMC8419389

[20] Pereira SM, Fonseca AM, Carvalho AS. Burnout in palliative care: a systematic review. Nurs Ethics. 2011;18(3):317–26. 21558108 10.1177/0969733011398092

[21] Gardiner C, Gott M, Ingleton C. Factors supporting good partnership working between generalist and specialist palliative care services: a systematic review. Br J Gen Pract. 2012;62(598):e353–362. 22546595 10.3399/bjgp12X641474 PMC3338057

[22] Hughes G, Shaw SE, Greenhalgh T. Why doesn’t integrated care work? Using strong structuration theory to explain the limitations of an english case. Sociol Health Illn. 2022;44(1):113–29. 34741766 10.1111/1467-9566.13398 PMC8936064

[23] Hughes MC, Vernon E, Hainstock A. The effectiveness of community-based palliative care programme components: a systematic review. Age Ageing 2023;52(9):afad175. 10.1093/ageing/afad175 PMC10517647 37740895

[24] Luckett T, Phillips J, Agar M, Virdun C, Green A, Davidson PM. Elements of effective palliative care models: a rapid review. BMC Health Serv Res. 2014;14:136. 24670065 10.1186/1472-6963-14-136 PMC3986907

[25] Boudy CA, Bouchez T, Caprini D, Pourrat I, Munck S, Barbaroux A. Home-based palliative care management: what are the useful resources for general practitioners? A qualitative study among gps in France. BMC Fam Pract. 2020;21(1):222. 33129251 10.1186/s12875-020-01295-7 PMC7603704

[26] Shipman C, Gysels M, White P, Worth A, Murray SA, Barclay S, Forrest S, Shepherd J, Dale J, Dewar S, et al. Improving generalist end of life care: National consultation with practitioners, commissioners, academics, and service user groups. BMJ. 2008;337:a1720. 18829640 10.1136/bmj.a1720 PMC2659492

[27] Bassi I, Pastorello S, Guerrieri A, Giancotti G, Cuomo AM, Rizzelli C, Coppola M, Valenti D, Nava S. Early palliative care program in idiopathic pulmonary fibrosis patients favors at-home and hospice deaths, reduces unplanned medical visits, and prolongs survival: A pilot study. Eur J Intern Med 2024;28:81–86. 10.1016/j.ejim.2024.05.024 38789287

[28] Temel JS, Greer JA, Muzikansky A, Gallagher ER, Admane S, Jackson VA, Dahlin CM, Blinderman CD, Jacobsen J, Pirl WF, et al. Early palliative care for patients with metastatic non-small-cell lung cancer. N Engl J Med. 2010;363(8):733–42. 20818875 10.1056/NEJMoa1000678

[29] Ferrell BR, Temel JS, Temin S, Alesi ER, Balboni TA, Basch EM, Firn JI, Paice JA, Peppercorn JM, Phillips T, et al. Integration of palliative care into standard oncology care: American society of clinical oncology clinical practice guideline update. J Clin Oncol. 2017;35(1):96–112. 28034065 10.1200/JCO.2016.70.1474

[30] Brighton LJ, Selman LE, Bristowe K, Edwards B, Koffman J, Evans CJ. Emotional labour in palliative and end-of-life care communication: A qualitative study with generalist palliative care providers. Patient Educ Couns. 2019;102(3):494–502. 30879492 10.1016/j.pec.2018.10.013

[31] Shimoinaba K, O’Connor M, Lee S, Kissane D. Nurses’ resilience and Nurturance of the self. Int J Palliat Nurs. 2015;21(10):504–10. 26505085 10.12968/ijpn.2015.21.10.504

[32] Mockli N, Denhaerynck K, De Geest S, Leppla L, Beckmann S, Hediger H, Zuniga F. The home care work environment’s relationships with work engagement and burnout: A cross-sectional multi-centre study in Switzerland. Health Soc Care Community. 2020;28(6):1989–2003. 32364334 10.1111/hsc.13010

[33] Heinen MM, van Achterberg T, Schwendimann R, Zander B, Matthews A, Kozka M, Ensio A, Sjetne IS, Moreno Casbas T, Ball J, et al. Nurses’ intention to leave their profession: a cross sectional observational study in 10 European countries. Int J Nurs Stud. 2013;50(2):174–84. 23107005 10.1016/j.ijnurstu.2012.09.019

[34] Jarrin OF, Kang Y, Aiken LH. Pathway to better patient care and nurse workforce outcomes in home care. Nurs Outlook. 2017;65(6):671–8. 28662969 10.1016/j.outlook.2017.05.009 PMC5712278

[35] Koh MYH, Hum AYM, Khoo HS, Ho AHY, Chong PH, Ong WY, Ong J, Neo PSH, Yong WC. Burnout and resilience after a decade in palliative care: what survivors have to teach Us. A qualitative study of palliative care clinicians with more than 10 years of experience. J Pain Symptom Manage. 2020;59(1):105–15. 31465787 10.1016/j.jpainsymman.2019.08.008

[36] Fattori A, Pedruzzi M, Cantarella C, Bonzini M. The burden in palliative care assistance: A comparison of psychosocial risks and burnout between inpatient hospice and home care services workers. Palliat Support Care. 2023;21(1):49–56. 35078551 10.1017/S1478951521001887

[37] Higashibata T, Hamano J, Nagaoka H, Sasahara T, Fukumori T, Arahata T, Kazama I, Maeno T, Kizawa Y. Work environmental factors associated with compassion satisfaction and end-of-life care quality among nurses in general wards, palliative care units, and home care settings: A cross-sectional survey. Int J Nurs Stud. 2023;143:104521. 37201336 10.1016/j.ijnurstu.2023.104521

[38] Bazata J, Meesters S, Bozzaro C, Handtke V, Schildmann J, Heckel M, Ostgathe C, Bausewein C, Schildmann E. SedPall study G: an easier way to die?-A qualitative interview study on specialist palliative care team members’ views on dying under sedation. Palliat Med. 2025;39(4):517–26. 39981842 10.1177/02692163251321320 PMC11977801

[39] Gillman L, Adams J, Kovac R, Kilcullen A, House A, Doyle C. Strategies to promote coping and resilience in oncology and palliative care nurses caring for adult patients with malignancy: a comprehensive systematic review. JBI Database Syst Rev Implement Rep. 2015;13(5):131–204. 10.11124/jbisrir-2015-1898 26455609

[40] Ingebretsen LP, Sagbakken M. Hospice nurses’ emotional challenges in their encounters with the dying. Int J Qual Stud Health Well-being. 2016;11:31170. 27258584 10.3402/qhw.v11.31170 PMC4891968

[41] Melvin CS. Historical review in Understanding burnout, professional compassion fatigue, and secondary traumatic stress disorder from a hospice and palliative nursing perspective. J Hospice Palliat Nurs. 2015;17(1):66–72.

[42] Ryde K, Hjelm K. How to support patients who are crying in palliative home care: an interview study from the nurses’ perspective. Prim Health Care Res Dev. 2016;17(5):479–88. 26932445 10.1017/S1463423616000037

[43] Petean M, Perciavalle M, Cerne D. Dilemmi etici nell’assistenza Ai pazienti terminali, comparazione Tra percezioni Degli infermieri e setting assistenziale. Studio Trasversale multicentrico. Rivista Italiana Di Cure Palliat. 2016;18(2):71–8.

[44] Hassankhani H, Rahmani A, Taleghani F, Sanaat Z, Dehghannezhad J. Palliative care models for cancer patients: learning for planning in nursing (Review). J Cancer Educ. 2020;35(1):3–13. 31020622 10.1007/s13187-019-01532-3

[45] Friesen L, Andersen E. Outcomes of collaborative and interdisciplinary palliative education for health care assistants: A qualitative metasummary. J Nurs Manag. 2019;27(3):461–81. 30194886 10.1111/jonm.12714

[46] Feldstain A. Psychosocial intervention in palliative care: what do psychologists need to know. J Health Psychol. 2024;29(7):707–20. 38282369 10.1177/13591053231222848 PMC11141107

[47] Kissane DW, Watson M, Breitbart W. Psycho-oncology in palliative and end of life care. Oxford University Press; 2022. p. 264.

[48] Sutherland R. Dying Well-Informed: the need for better clinical education surrounding facilitating End-of-Life conversations. Yale J Biol Med. 2019;92(4):757–64. 31866792 PMC6913833

[49] Leget C. The relation between cultural values, euthanasia, and spiritual care in the Netherlands. Pol Arch Intern Med. 2017;127(4):261–6. 28400548 10.20452/pamw.3979

[50] Liefbroer AI, Foppen A, Wierstra IR, Nagel I. The spiritual care intervention in dialogue with your life story: results of a longitudinal study on palliative clients’ spiritual wellbeing. Palliat Med. 2025;39(3):413–24. 39968917 10.1177/02692163251319143 PMC11877982

[51] De Vincenzo F, Lombardo L, Iani L, Maruelli A, Durante S, Ragghianti M, Park CL, Innamorati M, Quinto RM. Spiritual well-being, dignity-related distress and demoralisation at the end of life-effects of dignity therapy: a randomised controlled trial. BMJ Support Palliat Care. 2024;13(e3):e1238–48. 36702519 10.1136/spcare-2022-003696

[52] Kuosmanen L, Hupli M, Ahtiluoto S, Haavisto E. Patient participation in shared decision-making in palliative care - an integrative review. J Clin Nurs. 2021;30(23–24):3415–28. 34028923 10.1111/jocn.15866

[53] Svendsen SJ, Grov EK, Staats K. Patients’ experiences with shared decision-making in home-based palliative care - navigation through major life decisions. BMC Palliat Care. 2024;23(1):101. 38627710 10.1186/s12904-024-01434-2 PMC11022472

[54] Rabben J, Vivat B, Fossum M, Rohde GE. Shared decision-making in palliative cancer care: A systematic review and metasynthesis. Palliat Med. 2024;38(4):406–22. 38481012 10.1177/02692163241238384 PMC11025308

[55] Knaul FM, Farmer PE, Krakauer EL, De Lima L, Bhadelia A, Jiang Kwete X, Arreola-Ornelas H, Gomez-Dantes O, Rodriguez NM, Alleyne GAO, et al. Alleviating the access Abyss in palliative care and pain relief-an imperative of universal health coverage: the lancet commission report. Lancet. 2018;391(10128):1391–454. 29032993 10.1016/S0140-6736(17)32513-8

[56] Treglia E. The empathic abilities in nursing students: a longitudinal study. Clin Ter. 2020;171(6):e549–54. 33151255 10.7417/CT.2020.2271

[57] Fauchon C, Faillenot I, Quesada C, Meunier D, Chouchou F, Garcia-Larrea L, Peyron R. Brain activity sustaining the modulation of pain by empathetic comments. Sci Rep. 2019;9(1):8398. 31182760 10.1038/s41598-019-44879-9 PMC6558033

