# Supplementary material for: Palliative care perceptions and well-being in nurses across healthcare settings: a comparative study from Reggio Emilia, Northern Italy
Source: BMC Palliat Care. 2025 Oct 21;24:264. doi: 10.1186/s12904-025-01911-2 (PMC12539175; doi:10.1186/s12904-025-01911-2)
Supplement: Supplementary file 1 — Supplementary material 1. [file 12904_2025_1911_MOESM1_ESM.docx]

**Supplementary Methods: questionnaire**

**Palliative care: comparing perceptions of care settings between hospital, hospice, and home care providers**

**1. Which category matches your profession?**

- Hospital nurse
- Home nurse
- Hospice nurse

**2. In the field of palliative care, do you think (yes/no):**

- Can a newly-employed hospital nurse work independently in this field?
- Can a newly-employed home nurse work independently in this field?
- Are sufficient resources guaranteed in your department/sector to provide effective care?
- Can home care meet the demand for palliative care?

**3. The first time you faced a palliative emergency (massive hemorrhage, severe dyspnea crisis, spinal cord compression...), were you able to remain calm?**

- Yes
- No
- It has never happened to me

**4. Have you ever experienced excessive emotional involvement? (Thinking that what happened to the patient could happen to you or your loved ones...)**

- Yes
- No

**5. If you were the patient, what is one quality you would want from a palliative care nurse? Tell me in one word.**

**6. Considering your current career in palliative care, have you ever wondered if this is the right place for you now and in the future?**

- I believe this job is suitable for me, and I want to continue.
- I believe this job is NOT suitable for me, and I want to change.
- Sometimes I have thought about changing.
- I'm satisfied as it is.

**7. In your opinion, is home nursing care, with the joint access of the palliative care doctor and the general practitioner, adequate for end-of-life assistance?**

- Yes
- No

**If you answered no to the above question, please provide a brief explanation.**

**8. Have you ever experienced a delicate situation and felt alone in handling it?**

- Yes
- No

**9. Have you ever had a patient die in front of you?**

- Yes
- No

**10. Have you ever cried (for any reason) in front of a patient?**

- Yes
- No

**11. In your opinion, at what stage of the disease would you be more inclined to propose palliative care?**

- In the initial phase alongside active treatments
- When active treatments are no longer effective
- Once the terminal phase of the disease has been reached

**12. In your department/sector, how demanding do you find supporting the patient's family?**

- Excessive
- Very
- Quite
- Slightly
- Not at all

**13. Have you ever managed the imminent end of life of a patient together with one or more colleagues (nurses, OSS, doctors...) present with you?**

- Always
- Often
- Sometimes
- Rarely
- Never

**14. In your opinion, when palliative sedation is initiated, can it be linked to a possible shortening of the patient's life?**

- Yes
- No

**15. When you start palliative sedation, how much responsibility do you feel?**

- Excessive
- A lot
- Quite a bit
- Little
- None

**16. Do you feel adequately prepared to communicate with patients and families about suffering and approaching death?**

- Yes
- No

**17. Has your organization provided you with adequate and specific psychological support if needed?**

- Yes
- No

**18. After a shift, you often feel tired. Which of the following options reflects your state at the end of the workday?**

- I feel very tired both physically and mentally.
- I feel more physically tired.
- I feel more mentally tired.
- I don't feel particularly tired.

**19. If during a real palliative emergency (massive hemorrhage, severe dyspnea crisis, spinal cord compression...) you realize you need help for various reasons (assisting the family and/or patient, difficulty managing your emotions, clinical practice doubts...), what would you do?**

- Implement strategies to manage alone to avoid overloading my colleagues.
- Call a colleague for a phone consultation.
- Ask the colleague to come to where I am to help me.
- Other: ___

**Supplementary Table S1**. Results from asking nurses to describe palliative care in one word.

| **Word** | **N** |
| --- | --- |
| Empathy | 11 |
| Listening | 7 |
| Competence | 5 |
| Understanding | 3 |
| Welcoming | 2 |
| Presence | 1 |
| Humanity | 1 |
| Knowing how to be there | 1 |
| Being there | 1 |
| Time | 1 |
| Advocacy | 1 |
| (Listening) without judgment | 1 |
| Effectiveness | 1 |
| Reassurance | 1 |
| Dignity | 1 |
| Respect | 1 |
| Kindness | 1 |
| Personalization | 1 |
| Gentleness | 1 |
| Personalization | 1 |
